# Influence of interleukin-6 on the pharmacokinetics and pharmacodynamics of osimertinib in patients with non-small cell lung cancer

**DOI:** 10.1007/s00280-025-04772-x

**Published:** 2025-03-29

**Authors:** Hayato Yokota, Kazuhiro Sato, Sho Sakamoto, Yuji Okuda, Masahide Takeda, Yumiko Akamine, Katsutoshi Nakayama, Masatomo Miura

**Affiliations:** 1 Department of Pharmacy, Akita University Hospital, Akita, Japan; 2 Department of Internal Medicine Division of Respiratory Medicine, Akita University School of Medicine, Akita, Japan; 3 Department of Pharmacokinetics, Akita University Graduate School of Medicine, 1-1-1 Hondo, Akita, 010-8543 Japan

**Keywords:** Area under the plasma concentration–time curve, Interleukin-6, Osimertinib, Overall survival, Polymorphism, Trough concentration

## Abstract

**Purpose:**

The inflammatory cytokine interleukin (IL)-6 reduces the activity of drug metabolic enzymes and promotes tumor progression. We investigated the effect of IL-6 on the pharmacokinetics of osimertinib and the association between an *IL-6* polymorphism and clinical outcomes in 30 patients with non-small cell lung cancer (NSCLC).

**Methods:**

Osimertinib and IL-6 plasma concentrations were measured on day 15 after therapy initiation. The genotype of *IL-6* 1800796G > C was identified using polymerase chain reaction–restriction fragment length polymorphism. Risk factors affecting overall survival (OS) were assessed by Cox proportional hazard regression analysis.

**Results:**

The IL-6 concentration was significantly correlated with the osimertinib trough plasma concentration (*r* = 0.423, *P* = 0.020) and area under the plasma concentration–time curve (*r* = 0.420, *P* = 0.021). The IL-6 concentration was significantly higher in patients with the *IL-6* rs1800796G allele versus C/C genotype (*P* = 0.024). OS was significantly shorter in patients with the *IL-6* rs1800796G allele versus C/C genotype (median: 15.1 vs. 48.9 months, *P* = 0.005). Univariate and multivariate analyses indicated that the *IL-6* rs1800796G allele is an independent risk factor for OS (crude hazard ratio = 7.07; *P* = 0.014; adjusted hazard ratio = 6.38; *P* = 0.021).

**Conclusion:**

A higher IL-6 concentration was associated with reduced metabolic activity of osimertinib, leading to increased osimertinib exposure. As the IL-6 concentration was higher in NSCLC patients with the *IL-6* rs1800796G allele, it might be an independent prognostic factor for patients treated with osimertinib.

## Introduction

Osimertinib, a third-generation epidermal growth factor receptor (EGFR) tyrosine kinase inhibitor (EGFR-TKI), has become a first-line treatment for non-small cell lung cancer (NSCLC) with EGFR mutations. In the FLAURA trial, osimertinib prolonged not only progression-free survival (PFS) but also overall survival (OS) compared with first-generation EGFR-TKIs, such as gefitinib and erlotinib (PFS: 18.9 vs. 10.2 months; OS: 38.6 vs. 31.8 months) [1, 2]. Furthermore, osimertinib demonstrated efficacy in patients with EGFR T790M-positive NSCLC who experienced disease progression after EGFR-TKI therapy [3].

Because several TKIs show a correlation between the plasma exposure–response and exposure–toxicity relationships, clinicians need to adjust the dosage based on the plasma concentrations of TKIs [4]. Patients with a high trough plasma concentration (C_0_) of osimertinib (≥ 211 ng/mL) are reported to have longer PFS than are those with lower concentrations [5]. On the other hand, osimertinib concentrations > 259 ng/mL are reported to increase the risk of toxicity [6]. The steady-state osimertinib concentration shows high inter-individual variability, with the percent coefficient of variation of the area under the plasma concentration–time curve (AUC) ranging from 43.0 to 72.8% [7]. For thus inter-individual variability, polymorphisms in drug-metabolizing enzymes and drug efflux transporters can affect drug pharmacokinetics and help explain the variability in clinical outcomes [8]. Osimertinib is metabolized by the drug-metabolizing enzymes cytochrome P450 (CYP) 3A4 and CYP3A5 [9], and it is a substrate of ATP-binding cassette (ABC) transporters, including P-glycoprotein encoded by *ABCB1* and BCRP encoded by *ABCG2*. The mean osimertinib C_0_ is significantly lower in patients with the T/T genotype of *ABCB1* 3435 C > T than in those with the C allele, and *ABCB1* 3435 C > T and *AGCG2* 34G > A are risk factors for progression of central nervous system diseases [10]. On the other hand, in contrast to this result [10], we observed no significant differences in the osimertinib plasma concentration according to *ABC* transporter polymorphisms (*ABCB1* 3435 C > T, *ABCB1* 1236 C > T, *ABCB1* 2677G > T/A, *ABCG2* 421 C > A) [11]. Therefore, until now, the factors contributing to the inter-individual variability of the osimertinib plasma concentration have not been fully understood.

Inflammatory conditions are major regulators of drug-metabolizing enzymes and transporters [12], which affect drug clearance in cancer patients [13]. Because the inflammatory cytokine interleukin (IL)-6 decreases CYP3A4 metabolic activity [14], it may affect the pharmacokinetics and toxicity of osimertinib. IL-6 promotes tumorigenesis via the JAK/STAT signaling pathway [15]. IL-6 levels are increased in a wide range of cancers, including multiple myeloma, lung cancer, colorectal cancer, renal cell carcinoma, and cervical cancer [16]. Two single nucleotide polymorphisms (rs1800795G > C and rs1800796G > C) in the promoter region of *IL-6* are reportedly associated with alterations in the transcriptional activity of IL-6, thereby causing variability in the IL-6 concentration among individuals [17, 18]. *IL-6* polymorphisms are correlated with lung cancer risk [19], and the *IL-6* rs1800796G > C polymorphism increases cancer susceptibility in Asian populations [20]. However, the influence of the *IL-6* rs1800796G > C polymorphism on the therapeutic efficacy of osimertinib is not known.

In this study, we investigated the relationship between the IL-6 concentration and the pharmacokinetics of osimertinib in Japanese patients with NSCLC. In addition, we also examined the effect of the *IL-6* rs1800796G > C polymorphism on the clinical outcomes of NSCLC patients treated with osimertinib.

## Materials and methods

### Patients and protocols

Thirty Japanese patients with NSCLC who were treated with osimertinib (TAGRISSO; AstraZeneca K.K., Osaka, Japan) at Akita University Hospital between July 2016 and July 2023 were enrolled consecutively in this study. The recommended starting dose was 80 mg once daily. Patients from whom blood samples could not be obtained during the osimertinib administration period were excluded from this study. The study was conducted according to the principles of the Declaration of Helsinki. The study protocol was approved by the Ethics Committee of Akita University School of Medicine (approval number: 2826), and all patients provided written informed consent for participation in the study. Demographic data, biological data, and clinical events (disease progression or death) were collected retrospectively from medical records.

### Sample collection

Whole blood samples were collected just prior to (C_0_), and at 1, 2, 4, 6, 8, 12, and 24 h on day 15 after, osimertinib administration. Measurements of osimertinib C_0_ and the IL-6 level were performed at the same time. Plasma was separated from whole blood by centrifugation and stored at − 80 °C until analysis.

### Analytical methods

The plasma concentration of osimertinib was measured by high-performance liquid chromatography and the ultraviolet method [11]. The calibration curve generated for the osimertinib concentration in human plasma was linear in the concentration range of 10–1000 ng/mL. The coefficients of variation and accuracies of intra- and inter-day assays at the concentration range of 10–1000 ng/mL were less than 11.4% and within 10.2%, respectively. The limit of quantification of osimertinib was 10 ng/mL.

The plasma concentration of IL-6 was measured using the Quantikine^®^ enzyme-linked immunosorbent assay (R&D Systems, Minneapolis, MN, USA), following the manufacturer’s instructions.

### Genotyping

DNA was extracted from peripheral blood samples using the QIAamp Blood Mini Kit (Qiagen, Tokyo, Japan) and was stored at − 80 °C until analysis. Genotyping of the *IL-6* 1800795G > C and 1800796G > C polymorphisms was performed using polymerase chain reaction–restriction fragment length polymorphism [21]. Because the G/G, G/C, and C/C genotypes of the *IL-6* rs1800796G > C polymorphism were detected in 2 (6.7%), 13 (43.3%), and 15 (50.0%) patients, respectively, the patients were divided into two groups: G/G plus G/C (*n* = 15) and C/C (*n* = 15) [22]. On the other hand, the G/G, G/C, and C/C genotypes of the *IL-6* rs1800795G > C polymorphism were detected in 30 (100%), 0 (0%), and 0 (0%) patients, respectively; thus, this *IL-6* polymorphism was not present in our cohort.

### Clinical endpoints

In the evaluation of osimertinib efficacy, the primary endpoint was OS, defined as the time from osimertinib treatment initiation to death from any cause. The secondary endpoint was PFS, defined as the time from osimertinib treatment initiation to a documented progression event, either clinical or death from any cause.

### Statistical procedures

Descriptive data are expressed as numbers (%) and medians [interquartile range]. Spearman’s rank correlation was used to assess the correlations of continuous values between groups, and all results are expressed as correlation coefficients (*r* values). Pharmacokinetic analysis of osimertinib was conducted via the standard noncompartmental method using WinNonlin (Pharsight Co., Mountain View, CA, USA; version 5.2). The AUC_0 − 24_ was calculated using the linear trapezoidal rule. Regarding polymorphisms, the Mann–Whitney U test or Kruskal–Wallis test was used to determine differences in continuous values between two or among three groups, respectively. The Kaplan–Meier method was used to estimate OS and PFS distributions for each group, and the distributions were then compared between groups using the log-rank test. Explanatory variables were initially analyzed as risk factors for OS by univariate analyses, and then these variables were subjected to univariate and multivariate analyses using Cox proportional hazards regression models. The median values of age, weight, body mass index (BMI), IL-6 concentration, and osimertinib C_0_ were used as the cutoff values. Hazard ratios (HRs) and 95% confidence intervals (CIs) were calculated from the Cox models. Results with a *P* value < 0.05 were considered statistically significant. Statistical analysis was performed using IBM SPSS Statistics version 27.0 for Windows (SPSS IBM Japan Inc., Tokyo, Japan).

## Results

### Patient characteristics

The clinical characteristics of the patients are shown in Table 1. Of the 30 patients, 22 (73.3%) were female and 8 (26.7%) were male. Histologically, all patients had adenocarcinoma; 29 (96.7%) patients had stage IV disease at diagnosis and 1 (3.3%) patient stage IIIb. Exon 19 deletion and the exon 21 L858R mutation were observed in 20 (66.7%) and 10 (33.3%) patients, respectively. The G/G, G/C, and C/C genotypes of the *IL-6* rs1800796G > C polymorphism were identified in 2 (6.7%), 13 (43.3%), and 15 (50.0%) patients, respectively. The median (quartile 1–3) age, weight, and BMI were 71.5 (62.0–75.0) years, 53.1 (46.3–61.7) kg, and 22.0 (19.5–24.2) kg/m^2^, respectively. The median IL-6 concentration on day 15 after beginning therapy was 3.00 pg/mL (1.00–6.82 pg/mL). The median C_0_ and AUC_0-24_ of osimertinib were 227 ng/mL (122–314 ng/mL) and 7250 ng∙h/mL (4020–8988 ng∙h/mL), respectively.

**Table 1:** Clinical characteristics of patients treated with osimertinib

Characteristic	Number (%)
Sex (Female: Male)	22 (73.3%): 8 (26.7%)
Tumor histology (Adenocarcinoma: Other)	30 (100%): 0 (0%)
Stage (IV: IIIb)	29 (96.7%): 1 (3.3%)
EGFR mutations (Exon 19 deletion: Exon 21 L858R)	20 (66.7%): 10 (33.3%)
Osimertinib therapy (First-line: Second-line or later)	11 (36.7%): 19 (63.3%)
Smoking (Yes: No)	13 (43.0%): 17 (57.0%)
*IL-6* rs1800796G> C (G/G: G/C: C/C)	2 (6.7%): 13 (43.3%): 15 (50.0%)
	Median (quartile 1–3)
Age (years)	71.5 (62.0 - 75.0)
Body weight (kg)	53.1 (46.3 - 61.7)
Body mass index (kg/m2)	22.0 (19.5 - 24.2)
Laboratory parameters	
White blood cells (×10^3^/mm^3^)	5.6 (4.3 - 6.8)
Red blood cells (×10^4^/mm^3^)	416 (373 - 437)
Hemoglobin (g/dL)	12.3 (11.4 - 13.5)
Platelets (×10^4^/mm^3^)	254 (220 - 298)
Aspartate aminotransferase (IU/L)	25 (22 - 30)
Alanine aminotransferase (IU/L)	24 (20 - 29)
Serum albumin (g/dL)	3.8 (3.5 - 4.1)
Total bilirubin (mg/dL)	0.5 (0.4 - 0.8)
Serum creatinine (mg/dL)	0.65 (0.52 - 0.75)
IL-6 (pg/mL)	3.00 (1.00 - 6.82)
Osimertinib C_0_ (ng/mL)	227 (122 - 314)
Osimertinib AUC_0-24_ (ng h/mL)	7250 (4020 - 8988)

Data are numbers or medians (quartile 1–3)

### Association of the osimertinib concentration with the IL-6 level and rs1800796G > C polymorphism

**Fig. 1:**
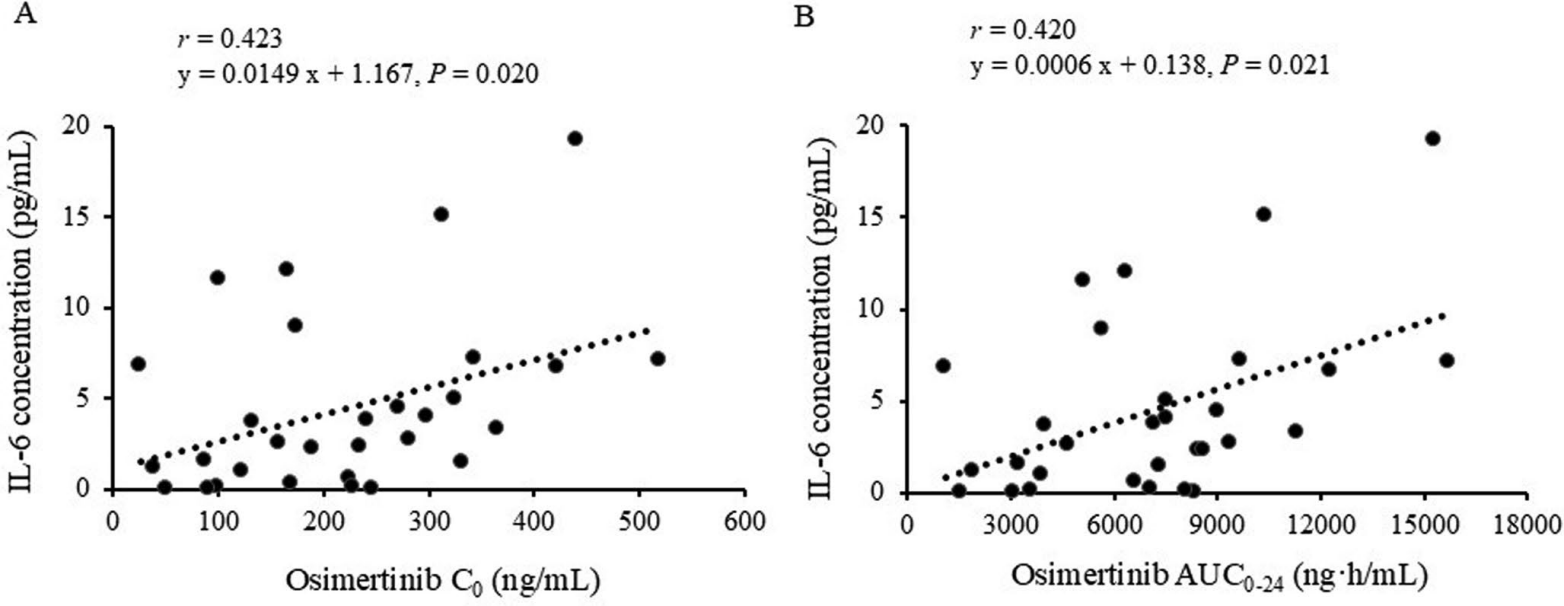
Correlation between the IL-6 level and osimertinib concentration. The trough plasma concentration (C_0_) (**A**) and AUC_0 − 24_ (**B**) of osimertinib

**Fig. 2:**
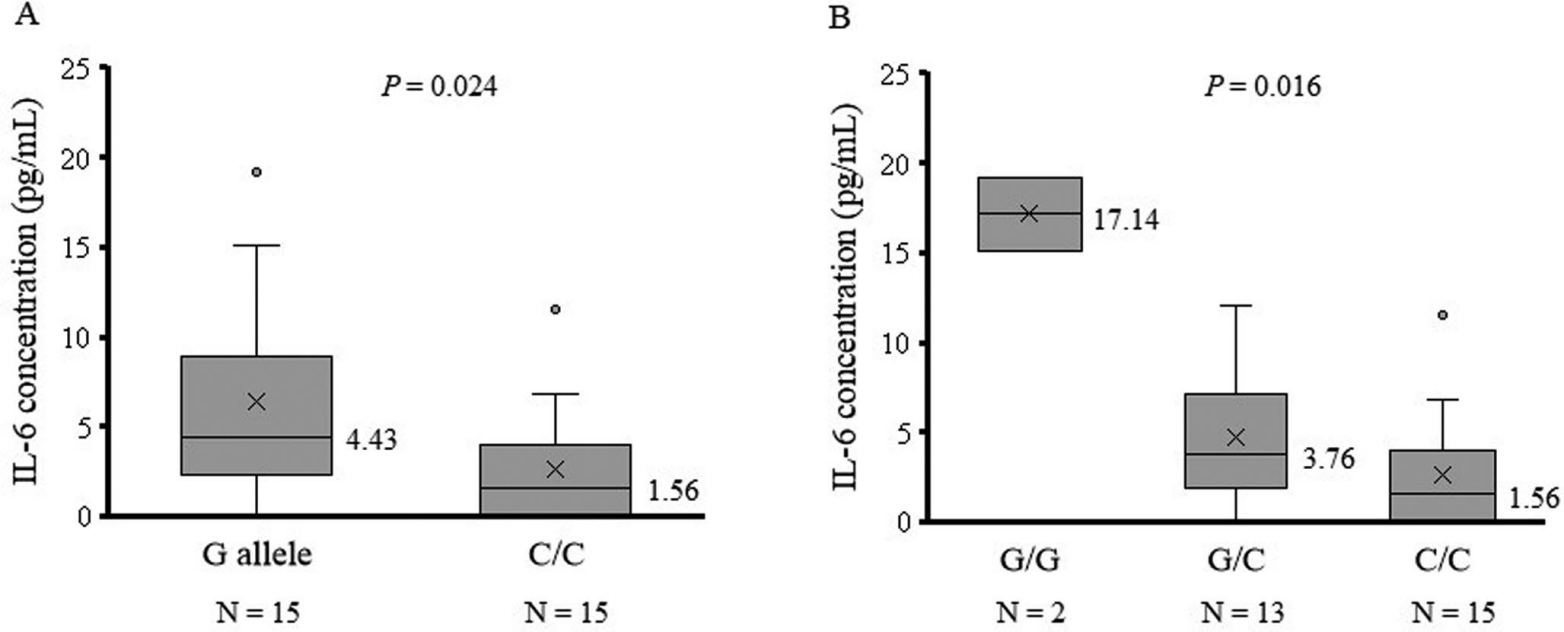
Influence of the *IL-6* rs1800796G > C polymorphism on the IL-6 level at 15 days after osimertinib therapy initiation. Comparisons of the IL-6 level (**A**) between the G allele and C/C genotype and (**B**) among the G/G, G/C, and C/C genotypes. The boxes represent the interquartile ranges, with the bold horizontal lines and numbers next to the boxes indicating the medians. The ends of each whisker (vertical lines) represent the smallest and largest values that were not outliers. Outliers (circles) are the values between 1.5 and 3 quartiles from the end of the box

A significant correlation was observed between the IL-6 concentration and osimertinib C_0_ (*r* = 0.423, *P* = 0.020) or AUC_0-24_ (*r* = 0.420, *P* = 0.021) (Fig. 1). The IL-6 concentration was significantly higher in patients with the *IL-6* rs1800796G allele than in those with the C/C genotype (median: 4.43 vs. 1.56 pg/mL, *P* = 0.024; Fig. 2A). There was a significant difference in the IL-6 concentration among the G/G, G/C, and C/C genotypes (median: 17.14 vs. 3.76 vs. 1.56 pg/mL, *P* = 0.016; Fig. 2B).

The C_0_ of osimertinib was significantly higher in patients with the *IL-6* rs1800796G allele compared with the C/C genotype (272 vs. 170 ng/mL, *P* = 0.044), whereas there was no significant difference in the AUC_0-24_ of osimertinib in patients with the *IL-6* rs1800796G allele versus C/C genotype, although the AUC_0-24_ tended to be higher in the former (8470 vs. 6593 ng∙h/mL, *P* = 0.065).

### Kaplan–meier curves for OS and PFS according to the IL-6 rs1800796G > C polymorphism

**Fig. 3:**
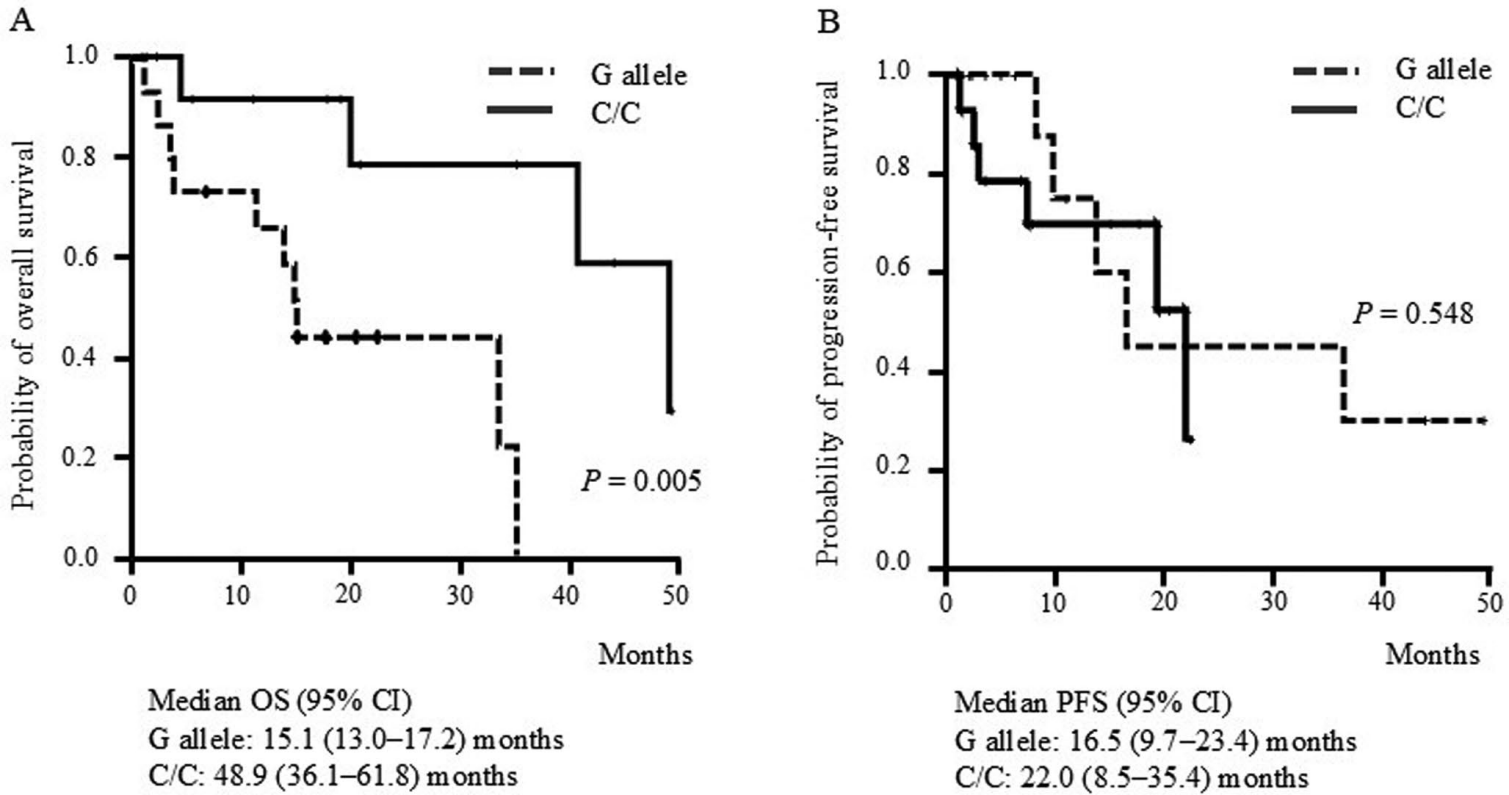
Kaplan–Meier curves for (**A**) overall survival (OS) and (**B**) progression-free survival (PFS) in patients with the *IL-6* rs1800796 G allele (dotted line) or C/C genotype (solid line)

As shown in Fig. 3A, the median OS times of patients with the *IL-6* rs1800796G allele and C/C genotype were 15.1 (95% CI: 13.0–17.2) and 48.9 (95% CI: 36.1–61.8) months, respectively, with a significantly shorter OS in the former than the latter patients (*P* = 0.005). On the other hand, no significant difference in PFS was observed between patients with the *IL-6* rs1800796G allele and those with the C/C genotype (median: 16.5 [95% CI: 9.7–23.4] vs. 22.0 [95% CI: 8.5–35.4] months, *P* = 0.548; Fig. 3B).

### Univariate and multivariate analyses of risk factors for OS

The results of the univariate and multivariate analyses using Cox proportional hazard regression models are shown in Table 2. The following candidate variables were evaluated: sex, age, body weight, BMI, EGFR mutation status, osimertinib therapy, smoking, *IL-6* rs1800796G > C polymorphism, IL-6 concentration, and osimertinib C_0_. Univariate analysis identified only the *IL-6* rs1800796G allele as a clinical risk factor for OS (crude HR [95% CI] for G allele vs. C/C: 7.07 [1.49–33.6], *P* = 0.014). In the Cox proportional hazards regression analysis adjusted for age, sex, and EGFR mutation status, the *IL-6* rs1800796G allele remained an independent factor influencing OS in NSCLC patients on osimertinib therapy (adjusted HR for G allele vs. C/C: 6.38 (95% CI: 1.32–30.6), *P* = 0.021).

**Table 2:** Univariate and multivariate analyses of risk factors for OS in patients treated with osimertinib

Variable		Univariate analysis		*P* value	Multivariate analysis		*P* value
		Crude HR (95% CI)			Adjusted HR^†^ (95% CI)		
Sex	Female	0.51	(0.16-1.62)	0.255			
	Male	1		-			
Age*	≥ 71.5	0.34	(0.10-1.11)	0.073			
	< 71.5	1		-			
Body weight*	≥ 53.1 kg	0.97	(0.32-2.91)	0.950			
	< 53.1 kg	1		-			
Body mass index*	≥ 22 kg/m^2^	1.40	(0.46-4.22)	0.554			
	< 22 kg/m^2^	1		-			
EGFR mutation status	Exon 19 deletion	1.19	(0.40-3.59)	0.751			
	Exon 21 L858R	1		-			
Osimertinib therapy	1st line	1.08	(0.28-4.22)	0.913			
	2nd line or later	1		-			
Smoking	Yes	1.82	(0.63-5.29)	0.27			
	No	1		-			
*IL-6* rs1800796G> C	G/G + G/C	7.07	(1.49-33.6)	0.014	6.38	(1.32-30.6)	0.021
	C/C	1		-	1		-
IL-6 concentration on day 15*	≥ 3 pg/mL	1.34	(0.43-4.20)	0.619			
	< 3 pg/mL	1		-			
Osimertinib C_0_ on day 15*	≥ 227 ng/mL	2.24	(0.74-6.77)	0.152			
	< 227 ng/mL	1		-			

*Cut-off values represent the medians. †Adjusted for age, sex, and EGFR mutations

## Discussion

The present study showed that the IL-6 concentration decreased the metabolic activity of osimertinib, leading to an increased plasma concentration of osimertinib. This is the first study to investigate the relationship between the IL-6 concentration and osimertinib pharmacokinetics in patients with EGFR mutation-positive NSCLC. Furthermore, we identified the G allele of the *IL-6* rs1800796G > C polymorphism as an independent prognostic factor for a poor clinical outcome in NSCLC patients treated with osimertinib.

The plasma concentration of osimertinib was affected by the IL-6 concentration. CYP3A4 was more sensitive to reduced enzyme activity induced by IL-6 compared with other CYPs, including CYP1A1, CYP2D6, and CYP3A5, in hepatocytes [23, 24]. In an in vitro study, pre-treatment of cultured cells with IL-6 reportedly decreased the expression of CYP3A4 protein and increased toxicity of gefitinib in a concentration-dependent manner [25]. Therefore, similar to an in vitro report of gefitinib [25], the plasma concentration of osimertinib may be increased in a concentration-dependent manner by IL-6. According to physiologically based pharmacokinetic models, the reductions in CYP3A enzyme activity from baseline under a constant level of IL-6 elevation (50–100 pg/mL) are predicted to be 40–52% in the liver and 41–54% in the gut [26]. In the present study, the degree of increase in the plasma concentration of osimertinib induced by inflammation was not evaluated. Therefore, further study is necessary.

We identified the *IL-6* rs1800796G > C polymorphism as an independent prognostic predictor of osimertinib clinical outcomes. The IL-6 protein concentration was higher in patients with lung cancer carrying the *IL-6* rs1800796G allele compared with the C/C genotype. This result supports previous reports of a higher IL-6 concentration in subjects with the G allele of the IL-6-634G > C polymorphism (rs1800796) [27, 28]. However, in the present study, the IL-6 concentration on day 15 after beginning osimertinib therapy was not an independent prognostic factor for OS. Our data should be interpreted with caution because the median IL-6 concentration early after osimertinib initiation was only 3.0 pg/mL, which is low. In a previous study, NSCLC patients had a higher median IL-6 concentration compared with healthy subjects (15.16 vs. 1.90 pg/mL) [29]. However, according to other reports, the IL-6 concentration was lower at baseline or before lung cancer progression than during disease progression in patients treated with osimertinib [30, 31]. Furthermore, the IL-6 concentration was well correlated with survival time after chemotherapy initiation, compared with before treatment, in NSCLC patients [32]. In our study, we performed measurements at an early time point after osimertinib initiation (day 15), and the IL-6 concentration is expected to increase after day 15 of treatment initiation. A previous report showed that low BMI (< 20 kg/m^2^) was associated with a shorter OS in osimertinib-treated patients [33]; however, BMI did not affect OS in our study. In contrast, the *IL-6* rs1800796G allele was still a significant predictor of poor OS after adjusting for sex, age, and EGFR mutation status in the multivariate analysis.

The present study did not identify the osimertinib C_0_ as a risk factor influencing clinical outcomes. No correlation was observed between the AUC at an osimertinib concentration of 20–240 mg and the probability of an objective response to osimertinib [34]. Previous studies showed that dividing patients into two groups based on an osimertinib C_0_ cut-off of 211 versus 226 ng/mL did not reveal a positive correlation between the C_0_ and OS; however, those studies did not take into consideration other factors that potentially influence OS [5, 6]. In NSCLC cells, IL-6 promotes metastasis by upregulating TIM-4 via the NF-κB pathway [35]. Furthermore, N-glycosylation-defective IL-6 promotes enhanced lung metastasis and osimertinib resistance via the SRC/YAP/SOX2 signaling pathway [36]. A higher IL-6 concentration was observed in osimertinib-resistant cells [30, 31], but the IL-6 inhibitor tocilizumab partially recovered this increase [37]. In patients treated with 80 mg osimertinib, the osimertinib C_0_ increased from before to after cancer progression [33]. Therefore, while a higher IL-6 concentration increased the plasma concentration of osimertinib, IL-6 is expected to contribute to drug resistance and tumor progression during long-term EGFR-TKI therapy.

## Conclusion

In conclusion, the IL-6 concentration affected the plasma concentration of osimertinib. Therefore, systemic inflammatory conditions may increase osimertinib exposure. Additionally, the *IL-6* rs1800796G allele was found to be associated with OS in patients with EGFR mutation-positive NSCLC. *IL-6* polymorphisms detected before osimertinib therapy may serve as useful prognostic predictors.

## Data Availability

No datasets were generated or analysed during the current study.

## Abbreviations

ABC: ATP-binding cassette
AUC: Area under the plasma concentration-time curve
BMI: Body mass index
C_0_: Trough plasma concentration
CI: Confidence interval
CYP: Cytochrome P450
EGFR: Epidermal growth factor receptor
HR: Hazard ratio
IL: Interleukin
NSCLC: Non-small cell lung cancer
OS: Overall survival
PFS: Progression-free survival
TKI: Tyrosine kinase inhibitor
